# Spontaneous Expulsion of a Large Submucosal Fibroid in the Postpartum Period: A Report of a Rare Case

**DOI:** 10.7759/cureus.114254

**Published:** 2026-08-10

**Authors:** Ei Mon Mon Kyaw, Ei Ei Phyo Naing, Myat Lae Pann

**Affiliations:** 1 Obstetrics and Gynaecology, Luton and Dunstable University Hospital, Luton, GBR; 2 Obstetrics and Gynaecology, Luton and Dunstable Hospital NHS Foundation Trust, Luton, GBR

**Keywords:** conservative management, gnrh agonist, hysteroscopy, leuprorelin acetate, postpartum involution, spontaneous myomectomy, submucosal leiomyoma, uterine leiomyoma

## Abstract

Spontaneous expulsion of uterine fibroids is an uncommon clinical event, most frequently described in the postpartum period or after uterine artery embolisation. Expulsion of a very large submucosal fibroid without surgical intervention is particularly rare. We report the case of a 39-year-old multiparous woman who was diagnosed with a large uterine fibroid during pregnancy. Following an uncomplicated term caesarean delivery, she developed persistent heavy menstrual bleeding that did not respond to initial medical treatment. Ultrasound assessment three months postpartum demonstrated a large submucosal fibroid measuring 116.5 × 92.5 × 100.7 mm, with significant distortion of the endometrial cavity. After counselling regarding medical and surgical options, she opted for conservative management and received leuprorelin acetate. Two months later, she spontaneously expelled a large mass per vagina without significant pain, haemorrhage or infection. Histopathological examination confirmed an infarcted leiomyoma with no evidence of malignancy. Serial ultrasound follow-up demonstrated a marked reduction in residual fibroid tissue, and subsequent diagnostic hysteroscopy confirmed a normal uterine cavity with no residual fibroid or intrauterine adhesions. Endometrial biopsy showed inactive endometrium with no hyperplasia, endometritis or malignancy. This case highlights spontaneous expulsion of a very large submucosal fibroid as a rare but recognised phenomenon in the postpartum period. Postpartum uterine involution and altered vascular dynamics may have played a key role, while gonadotropin-releasing hormone agonist therapy may have acted as a contributing factor rather than the primary cause. Careful follow-up is essential to confirm complete resolution and exclude residual pathology.

## Introduction

Uterine fibroids (leiomyomas) are the most common benign tumours of the female reproductive tract, with a cumulative incidence of up to 60% by the age of 35 years and over 80% by 50 years in some populations [1,2]. Although many fibroids are asymptomatic, their clinical presentation depends largely on size, number and anatomical location. Submucosal fibroids, which distort the endometrial cavity, are particularly associated with heavy menstrual bleeding, anaemia, subfertility and adverse reproductive outcomes, and are therefore more likely to require intervention [3,4].

Management options for uterine fibroids include expectant, medical and surgical approaches, tailored to symptom severity, reproductive wishes and patient preference [4,5]. Surgical treatment, including myomectomy or hysterectomy, remains definitive but is associated with operative risks and potential impact on fertility. Consequently, medical therapies such as gonadotropin-releasing hormone (GnRH) agonists are frequently used to reduce fibroid size and control symptoms, either as a temporising measure or as an adjunct to surgery [6-10].

Spontaneous expulsion of uterine fibroids is an uncommon clinical phenomenon. It has most frequently been reported in association with the postpartum period or following uterine artery embolisation, where uterine involution, altered vascular supply and increased myometrial contractility may contribute to fibroid degeneration and detachment [11-15]. In contrast, spontaneous expulsion in the absence of direct procedural intervention is rare, particularly for large fibroids.

Although a small number of case reports have described fibroid expulsion during GnRH agonist therapy, these cases are uncommon and typically involve smaller or pedunculated lesions, and the causal relationship remains uncertain [16-19]. The interplay between postpartum physiological changes and medical therapy in facilitating fibroid expulsion is not well understood.

We report a rare case of spontaneous expulsion of a very large submucosal fibroid in the postpartum period, with possible contribution from GnRH agonist therapy, resulting in complete resolution without surgical intervention.

## Case presentation

**Figure 1:**
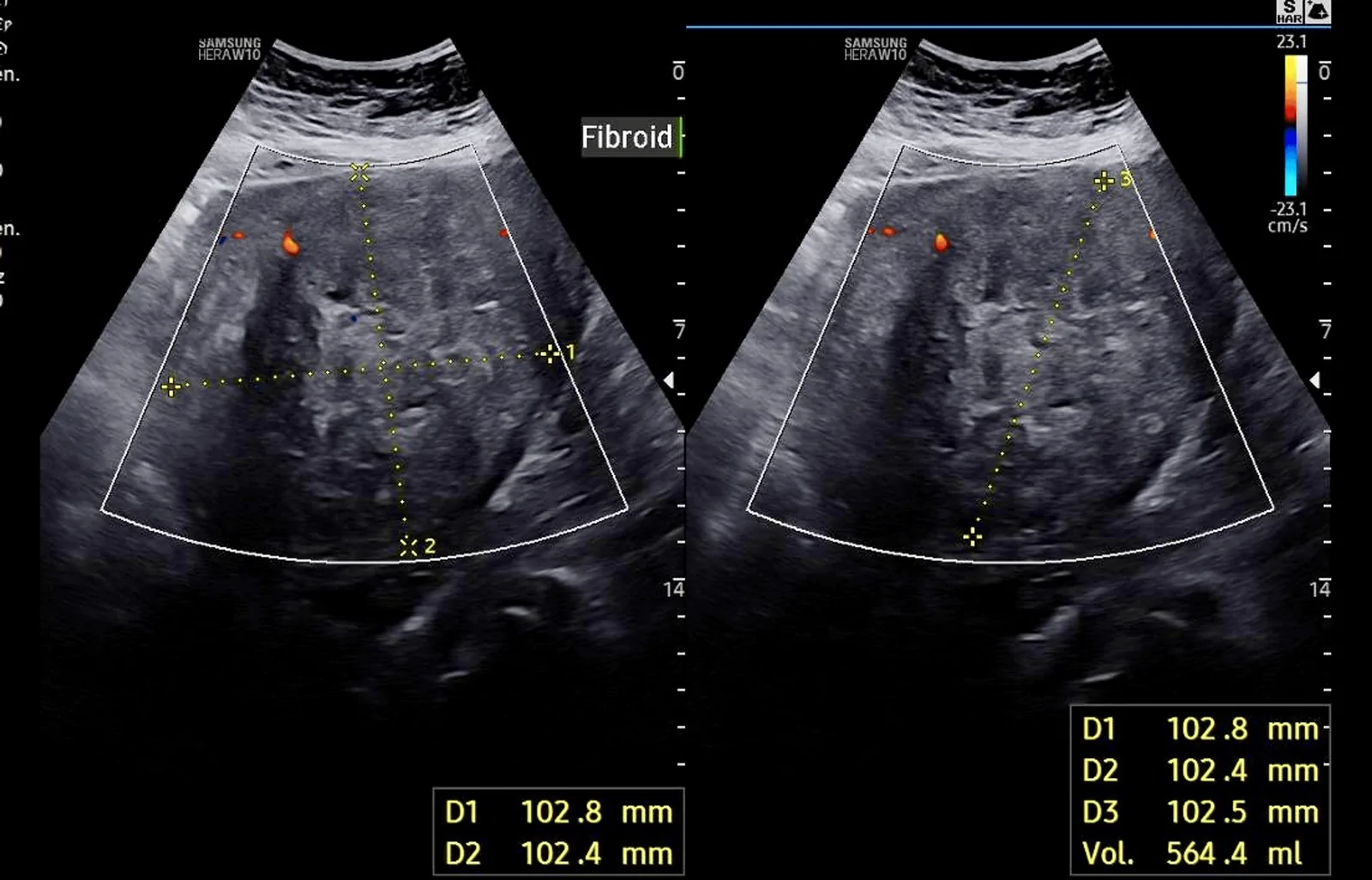
Early pregnancy ultrasound demonstrating a large uterine fibroid measuring approximately 102.8 × 102.4 × 102.5 mm.

A 39-year-old pregnant lady with three previous vaginal deliveries was diagnosed with a large uterine fibroid during her fourth pregnancy. Early pregnancy ultrasound at six weeks' gestation demonstrated a large uterine fibroid measuring approximately 102.8 × 102.4 × 102.5 mm (Figure 1). Subsequent antenatal imaging showed an interval increase in fibroid size, measuring 139 × 118 × 152 mm at 16 weeks’ gestation, located in the left anterior uterine wall.

**Figure 2:**
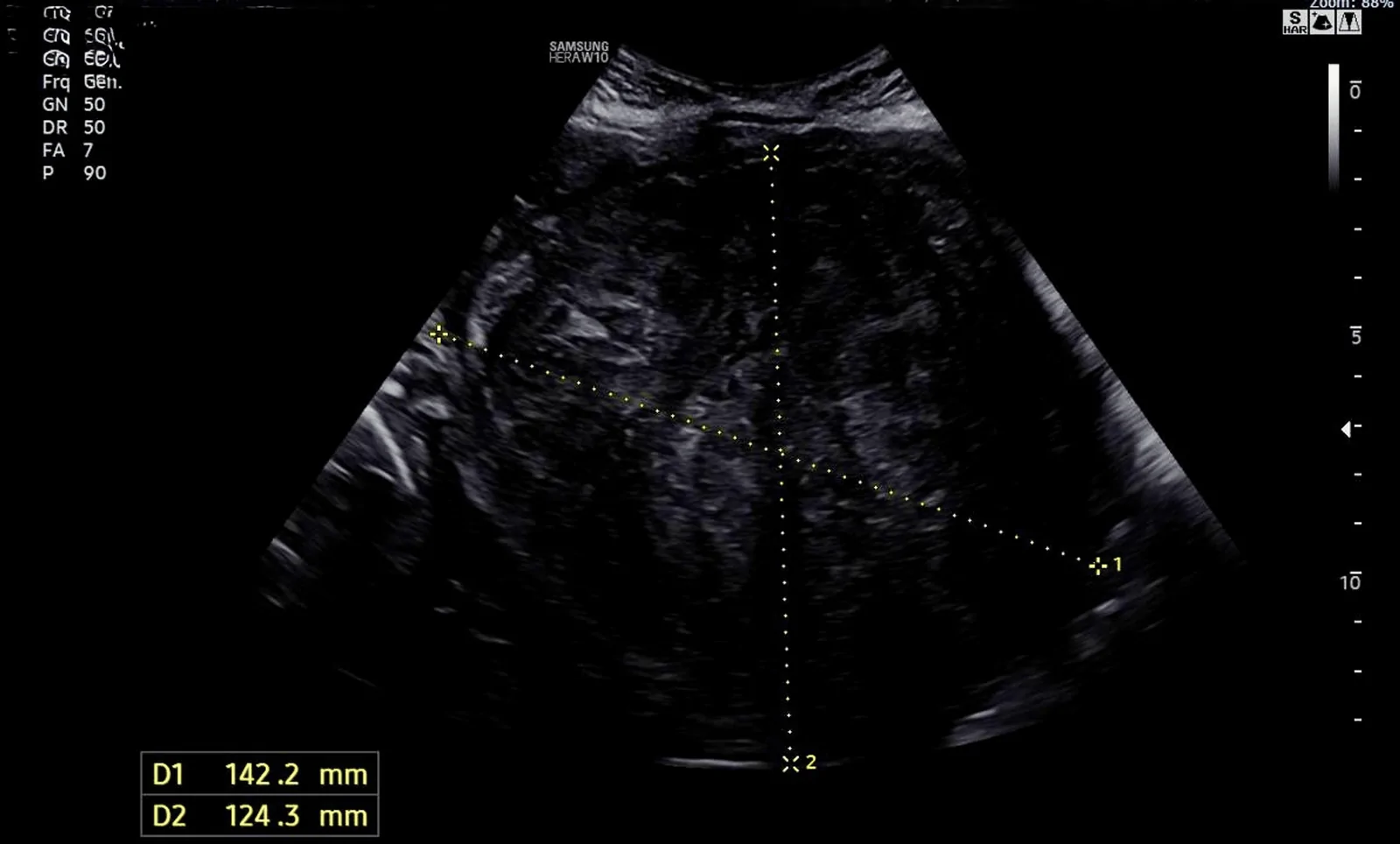
Anomaly scan ultrasound demonstrating a large uterine fibroid measuring approximately 142.2 × 124.3 mm.

Anomaly scan imaging again demonstrated a large fibroid, measuring approximately 142.2 × 124.3 mm (Figure 2). No additional uterine pathology was identified. The remainder of the pregnancy was uncomplicated, with no antenatal pain, bleeding, or preterm labour.

She underwent an elective lower-segment caesarean section at term for persistent breech presentation. Intraoperatively, a large anterior uterine fibroid was visualised and left in situ. The procedure was complicated by an estimated blood loss of 1.1 litres, although the patient remained haemodynamically stable and did not require blood transfusion.

**Figure 3:**
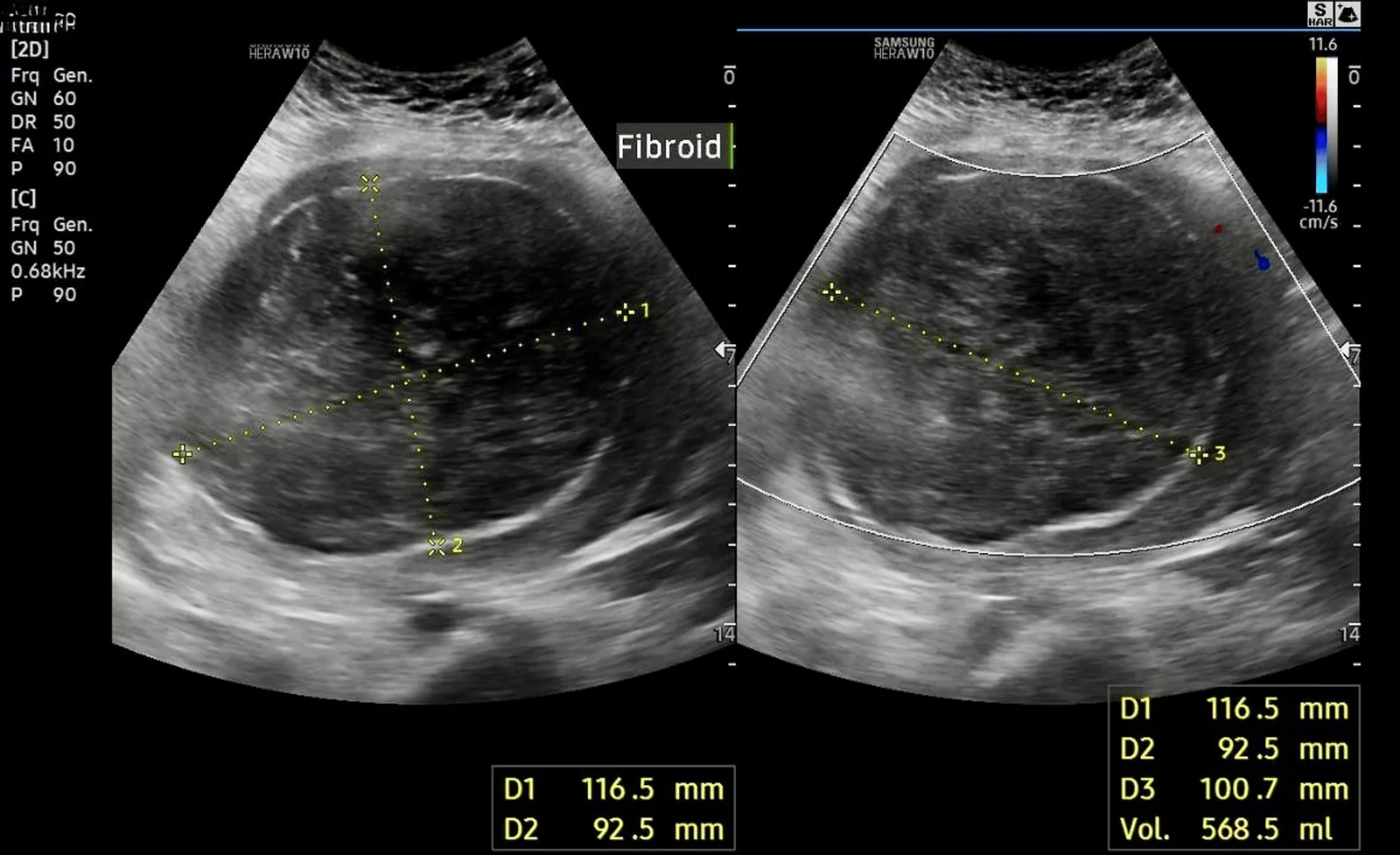
Three-month postpartum transabdominal ultrasound demonstrating a large submucosal fibroid measuring 116.5 × 92.5 × 100.7 mm, with significant distortion of the endometrial cavity. No clear sonographic evidence of retained products of conception was identified, and the endometrial stripe could not be reliably measured due to cavity distortion by the fibroid.

Following delivery, the patient reported persistent heavy menstrual bleeding that significantly affected her quality of life. For this reason, oral progestogen was prescribed in primary care as initial medical management. This was ineffective, and she presented to the gynaecology emergency clinic at 12 weeks postnatally for further assessment. Secondary postpartum haemorrhage and retained products of conception were considered as part of the differential diagnosis. Abdominal examination revealed a uterus enlarged to approximately 26-week gestational size. Subsequent transabdominal ultrasound demonstrated a large submucosal fibroid measuring 116.5 × 92.5 × 100.7 mm, with significant distortion of the endometrial cavity. No clear sonographic evidence of retained products of conception was identified (Figure 3).

After comprehensive counselling regarding the available management options, including definitive surgical treatment with myomectomy or hysterectomy, the patient was planned for surgical management. In view of the large fibroid size, persistent bleeding symptoms, and anticipated operative complexity, GnRH agonist therapy was commenced as a preoperative temporising measure to reduce bleeding, improve symptoms, and potentially reduce fibroid vascularity and size before surgery. She received leuprorelin acetate 11.25 mg, planned as two doses administered three months apart. Following the first dose, she reported a marked improvement in menstrual bleeding and a reduction in pelvic pressure symptoms.

Unexpectedly, approximately two months after the initial leuprorelin injection and before the second dose or planned surgical intervention, she spontaneously passed a large tissue mass per vagina. This was not associated with significant pain, haemorrhage, or infection. Histopathological examination demonstrated an entirely infarcted spindle cell lesion with ghost outlines of spindle cells. Although extensive infarction limited definitive assessment, the appearances were considered most in keeping with an infarcted smooth muscle tumour, consistent with an infarcted leiomyoma in the clinical context. Given the subsequent residual fibroid tissue seen on imaging, this was interpreted as spontaneous expulsion of the predominantly intracavitary component, followed by regression and calcification of the residual intramural component (Table 1).

**Table 1:** Histopathological findings of the spontaneously expelled tissue mass. The table summarises the macroscopic and microscopic findings of the tissue mass passed vaginally. The findings were most consistent with an infarcted smooth muscle tumour, consistent with an infarcted leiomyoma in the clinical context.

Component	Finding
Specimen	Soft tissue mass passed vaginally
Macroscopy	Brown-green tissue mass covered with yellowish exudate; smooth, pink, homogeneous cut surface on slicing measured 80 × 19 × 20 mm
Microscopy	Entirely infarcted lesion with ghost outlines of spindle cells
Interpretation	Appearance not fully diagnostic due to extensive infarction, but most in keeping with an infarcted smooth muscle tumour
Diagnosis	Infarcted spindle cell lesion, consistent with infarcted leiomyoma in the clinical context

**Figure 4:**
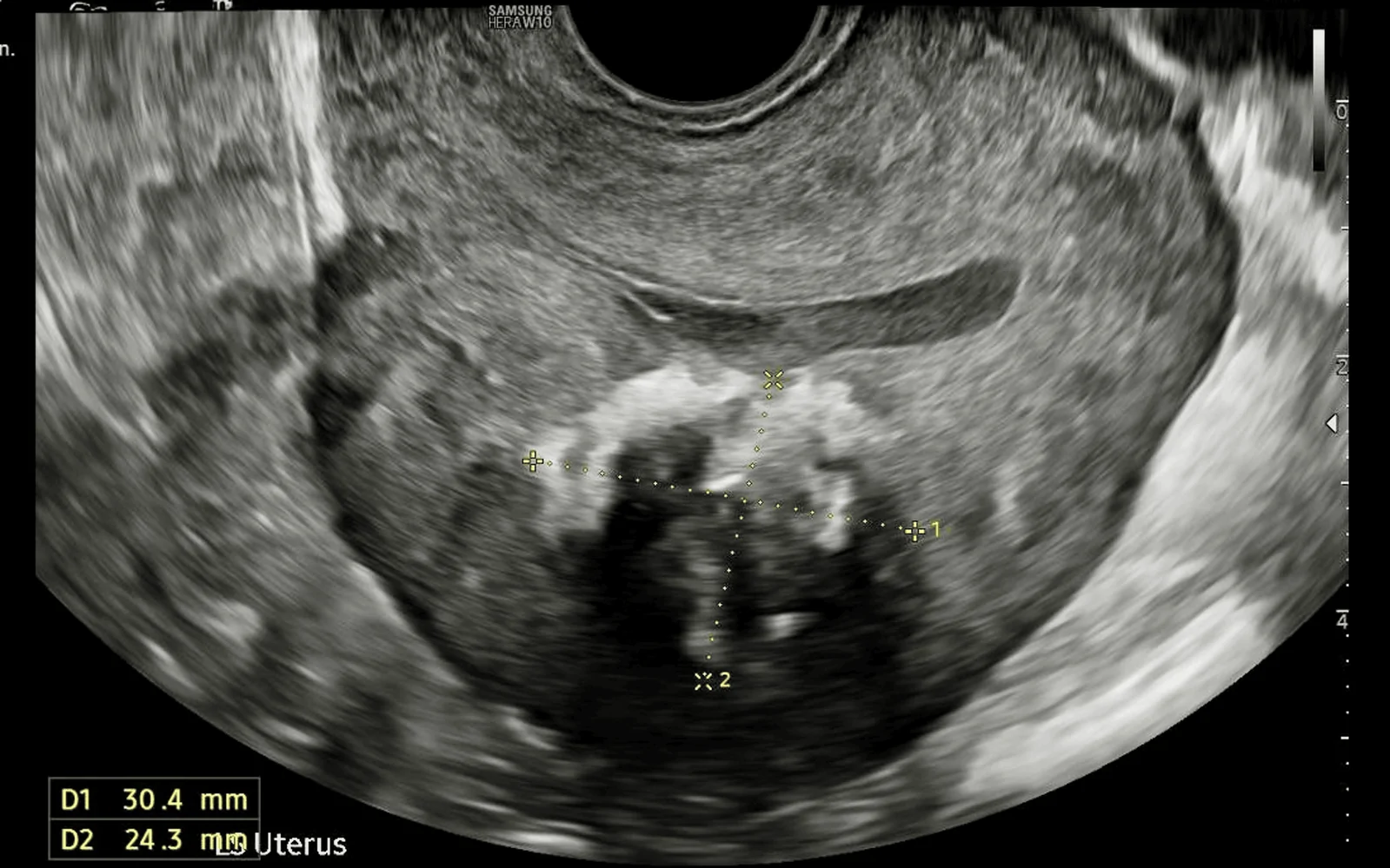
Follow-up ultrasound after spontaneous fibroid expulsion showing marked interval reduction. Follow-up ultrasound performed one month after spontaneous vaginal expulsion of the fibroid, demonstrating marked interval reduction in size, with a residual anterior fibroid adjacent to the endometrial cavity measuring approximately 30 × 24 × 34 mm.

A follow-up ultrasound performed one month after spontaneous expulsion demonstrated marked interval reduction in fibroid size, with a residual anterior submucosal fibroid measuring approximately 30 × 24 × 34 mm (Figure 4). The case was discussed at a multidisciplinary team meeting, and interval reassessment with repeat imaging and diagnostic hysteroscopy was recommended to evaluate for residual disease and assess endometrial cavity integrity.

**Figure 5:**
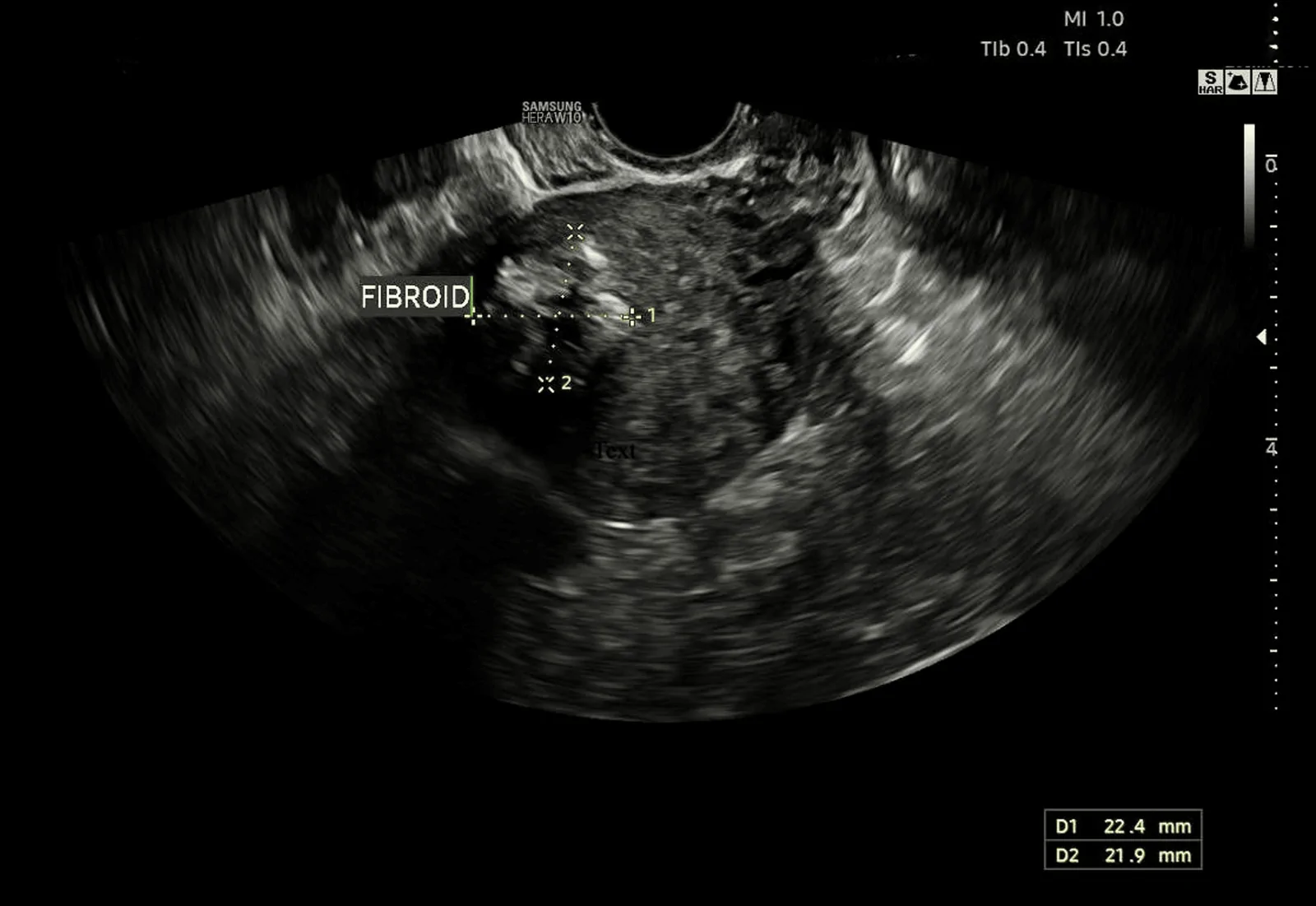
Final follow-up ultrasound showing a small calcified intramural fibroid. Subsequent follow-up transvaginal ultrasound performed three months after the previous scan, demonstrating a small calcified intramural fibroid measuring approximately 22 × 21 mm adjacent to the endometrial cavity.

A subsequent ultrasound performed three months later demonstrated a small calcified intramural fibroid measuring approximately 22 × 21 mm, located adjacent to the endometrial cavity, with a clearly defined endometrial thickness of 5.7 mm. Overall, the interval ultrasound findings demonstrated marked reduction in fibroid size following spontaneous expulsion, with subsequent calcification of the small residual intramural component adjacent to the endometrial cavity (Figure 5).

Diagnostic hysteroscopy performed later that month revealed a normal uterine cavity, with no residual intracavitary fibroid or intrauterine adhesions. Follow-up endometrial biopsy demonstrated inactive endometrium, with no evidence of chronic endometritis, hyperplasia, or malignancy (Table 2). As the patient remained asymptomatic and all follow-up investigations were reassuring, she was discharged from gynaecology follow-up.

**Table 2:** Histopathological findings of the expelled tissue mass and follow-up endometrial biopsy. This table summarises the histopathological findings of the spontaneously expelled tissue mass and subsequent follow-up endometrial biopsy. The expelled tissue showed extensive infarction, limiting definitive assessment, but the appearances were most in keeping with an infarcted smooth muscle tumour in the clinical context. Follow-up endometrial biopsy was reassuring, with no evidence of chronic endometritis, hyperplasia, or malignancy.

Specimen	Macroscopic findings	Microscopic findings	Interpretation
Expelled vaginal tissue mass	Brown-green soft tissue mass covered with yellowish exudate; smooth, pink, homogeneous cut surface on slicing	Entirely infarcted lesion with ghost outlines of spindle cells	Appearances were most in keeping with an infarcted smooth muscle tumour, consistent with an infarcted leiomyoma in the clinical context
Endometrial biopsy	Two cream tissue fragments measuring 3 mm and 6 mm	Inactive endometrium; no chronic endometritis, hyperplasia, or malignancy	Reassuring follow-up biopsy with no evidence of malignancy

A comparison with previously published cases of postpartum fibroid expulsion or regression and fibroid expulsion associated with GnRH therapy is summarised in Table 3.

**Table 3:** Clinical timeline of diagnosis, treatment, spontaneous fibroid expulsion and follow-up. This table summarises the chronological clinical course from antenatal diagnosis through postpartum symptoms, preoperative GnRH agonist therapy, spontaneous expulsion of the predominantly intracavitary component, and subsequent imaging, hysteroscopic and histological follow-up. GnRH: gonadotropin-releasing hormone

Time point	Clinical event or finding
Early pregnancy	Large uterine fibroid identified antenatally
Term pregnancy	Elective lower-segment caesarean section for persistent breech presentation; fibroid left in situ
Postpartum period	Persistent heavy menstrual bleeding affecting quality of life
Nine weeks postpartum	Oral progestogen prescribed in primary care as initial medical management
12 weeks postpartum	Gynaecology emergency clinic review; uterus enlarged to approximately 26-week size
12 weeks postpartum	Ultrasound showed large submucosal fibroid measuring 116.5 × 92.5 × 100.7 mm, with distortion of the endometrial cavity
After counselling	Planned surgical management; leuprorelin acetate 11.25 mg commenced as preoperative temporising therapy
Approximately two months after leuprorelin	Spontaneous passage of a large tissue mass per vagina
Post-expulsion histology	Infarcted spindle cell lesion, most in keeping with infarcted smooth muscle tumour in the clinical context
One month after expulsion	Ultrasound showed residual anterior fibroid measuring approximately 30 × 24 × 34 mm
Three months later	Ultrasound showed a small calcified intramural fibroid measuring approximately 22 × 21 mm with endometrial thickness 5.7 mm
Final follow-up	Hysteroscopy showed a normal uterine cavity with no residual intracavitary fibroid; biopsy showed inactive endometrium with no malignancy

## Discussion

Spontaneous expulsion of uterine fibroids is a rare but recognised clinical occurrence. It has most commonly been described in the postpartum period and following uterine artery embolisation, where physiological and vascular changes contribute to fibroid degeneration, detachment and eventual expulsion [12-15]. In contrast, expulsion occurring in the absence of direct procedural intervention remains uncommon, particularly for large fibroids.

The postpartum period represents a unique physiological state in which uterine involution, reduced uterine blood flow and increased myometrial contractility may significantly influence fibroid behaviour. These changes can result in ischaemia, degeneration and, in some cases, migration of fibroids toward the endometrial cavity. Some case reports have described spontaneous regression or expulsion of fibroids following delivery, supporting the role of postpartum uterine remodelling as a key contributing mechanism [11-15]. In the present case, the fibroid, initially intramural during pregnancy, evolved into a predominantly submucosal lesion postpartum, suggesting dynamic structural changes that may have facilitated its eventual expulsion. Based on its postpartum relationship to the endometrial cavity, the lesion was considered most consistent with a submucosal fibroid, likely International Federation of Gynaecology and Obstetrics (FIGO) type 1-2. However, precise FIGO classification was limited by marked distortion of the endometrial cavity and the absence of MRI assessment.

From an imaging perspective, the differential diagnosis for postpartum heavy menstrual bleeding with an enlarged uterus includes retained products of conception, postpartum haematoma, adenomyosis, degenerating fibroid, and, rarely, malignant uterine pathology. Retained products of conception were considered clinically; however, ultrasound demonstrated a well-defined large fibroid mass with marked distortion of the endometrial cavity and no clear intracavitary echogenic or vascular tissue suggestive of retained products. Postpartum haematoma was considered less likely given the chronic clinical course and serial imaging appearance. Adenomyosis was not supported by the presence of a dominant circumscribed mass lesion. Although sarcomatous change cannot be excluded by ultrasound alone, the subsequent spontaneous expulsion, histopathological findings most in keeping with an infarcted smooth muscle tumour, normal hysteroscopic cavity assessment, reassuring endometrial biopsy, clinical improvement, and endometrial cavity clearance supported a benign process.

Although GnRH agonists are well established in reducing fibroid size and improving symptoms [6-10], their role in fibroid expulsion remains less clearly defined. GnRH agonists induce a hypo-oestrogenic state that reduces fibroid vascularity and promotes cellular apoptosis, potentially leading to ischaemic degeneration [6-10]. In submucosal or predominantly intracavitary fibroid components, such changes may weaken the attachment between the fibroid and the underlying myometrium, thereby facilitating separation. A limited number of case reports have described fibroid expulsion during GnRH agonist therapy; however, these typically involve smaller or pedunculated lesions [16-19].

Postpartum uterine involution, reduced uterine blood flow, fibroid infarction, and myometrial contractility were likely the predominant mechanisms in this case. This is supported by the recognised occurrence of fibroid expulsion in the postpartum period and after uterine artery embolisation, where vascular occlusion and subsequent ischaemic degeneration are central mechanisms. The temporal association with leuprorelin acetate is noteworthy; however, causality cannot be established from a single case. Leuprorelin may have reduced fibroid vascularity and promoted degeneration, but it cannot be determined whether expulsion would have occurred without GnRH agonist therapy.

The exceptionally large size of the fibroid in this case- exceeding 11 cm - makes spontaneous expulsion particularly unusual. Most reported cases involve significantly smaller lesions, and large fibroids are generally managed surgically due to concerns regarding persistent symptoms, complications, and incomplete resolution [3,5]. In the present case, definitive surgical treatment was initially planned because of the fibroid size, persistent bleeding symptoms, and significant distortion of the endometrial cavity. GnRH agonist therapy was used as a preoperative temporising measure rather than as definitive conservative treatment. The subsequent spontaneous expulsion was therefore unexpected and altered the clinical course, allowing surgery to be avoided after reassuring post-expulsion assessment. A comparison with previously published cases of postpartum fibroid expulsion or regression and fibroid expulsion associated with GnRH therapy is summarised in Table 4.

**Table 4:** Comparison of previously reported cases with the present case. This table summarises selected previously reported cases of spontaneous fibroid expulsion or regression in the postpartum period and cases associated with GnRH therapy. Compared with earlier reports, the present case is notable for the very large postpartum fibroid size, planned surgical management with leuprorelin acetate used as preoperative temporising therapy, unexpected spontaneous vaginal expulsion before planned surgery, and comprehensive follow-up with serial ultrasound, histopathology, hysteroscopy, and endometrial biopsy. GnRH: gonadotropin-releasing hormone

Author, year	Clinical context	Fibroid type/location	Treatment or exposure	Outcome	Relevance to present case
Honoré and Reid, 1985 [12]	Postpartum period	Uterine leiomyoma	Conservative	Uncomplicated spontaneous postpartum expulsion	Supports postpartum expulsion as a recognised but rare event
Haskins et al., 2001 [13]	Pregnancy with postpartum change	Intramural leiomyoma becoming pedunculated postpartum	Conservative	Postpartum pedunculated transformation	Demonstrates dynamic fibroid anatomical change after delivery
De Cure et al., 2013 [11]	Non-embolisation case	Large submucosal uterine fibroid	Conservative	Spontaneous fibroid degeneration and expulsion	Supports spontaneous expulsion without uterine artery embolisation
Kim, 2018 [14]	Postpartum after vaginal delivery	Large uterine fibroid	Conservative management	Spontaneous complete regression after delivery	Supports postpartum uterine involution as a possible mechanism for fibroid regression
Zhang et al., 2018 [15]	Postpartum after caesarean delivery	Huge cervical leiomyoma	Conservative/postpartum observation followed by vaginal expulsion	Spontaneous expulsion from the vagina after caesarean delivery	Similar postpartum and post-caesarean context, but cervical rather than submucosal/intramural location
Yu et al., 2001 [16]	GnRH agonist therapy	Large submucosal leiomyoma	GnRH agonist	Spontaneous expulsion/protrusion through the introitus	Supports possible association between GnRH agonist therapy and expulsion of submucosal fibroids
Wen et al., 2006 [17]	Long-acting GnRH agonist therapy	Submucosal myoma	Long-acting GnRH agonist	Vaginal expulsion with subsequent ultrasound showing no residual submucosal myoma	Supports possible expulsion during GnRH agonist treatment
Atilgan et al., 2013 [18]	GnRH agonist therapy	Myoma	GnRH agonist	Spontaneous myomectomy	Further supports rare reports of fibroid expulsion after GnRH agonist exposure
Ishizawa et al., 2020 [19]	GnRH antagonist therapy	Pedunculated submucosal leiomyoma	GnRH antagonist	Prolapse through the cervix during treatment	Shows a related mechanism of medical therapy-associated leiomyoma prolapse
Present case	Postpartum after caesarean delivery with GnRH agonist therapy	Very large postpartum submucosal fibroid, preceded by large antenatal intramural/anterior fibroid	Conservative management with leuprorelin acetate	Spontaneous vaginal expulsion, marked interval reduction on ultrasound, normal hysteroscopic cavity, and reassuring endometrial biopsy	Notable for very large postpartum size, conservative management, serial ultrasound documentation, histopathology, hysteroscopy, and complete clinical resolution

Compared with previously reported cases, the present case is notable for the very large postpartum fibroid size, planned surgical management with preoperative GnRH agonist therapy, unexpected spontaneous vaginal expulsion before surgery, and comprehensive follow-up with serial ultrasound, histopathology, hysteroscopy, and endometrial biopsy. Importantly, spontaneous fibroid expulsion may be associated with complications including acute pain, heavy bleeding or infection, and clinicians should remain vigilant. Comprehensive post-expulsion evaluation is essential to confirm complete resolution and exclude retained tissue, intrauterine adhesions or endometrial pathology. In this case, serial imaging and diagnostic hysteroscopy confirmed complete clearance of the uterine cavity and restoration of normal endometrial architecture.

Overall, this case supports the concept that spontaneous expulsion of submucosal fibroids, although rare, can occur as a result of multifactorial processes, particularly in the postpartum setting [11-15]. Medical therapy may act as an adjunctive factor rather than a primary cause [16,18,19]. Recognition of this possibility may help inform counselling and management decisions in selected patients seeking to avoid surgical intervention.

## Conclusions

Spontaneous expulsion of a large submucosal fibroid is a rare but recognised clinical event, particularly in the postpartum setting. This case demonstrates spontaneous expulsion of the predominantly intracavitary component of a very large postpartum submucosal fibroid, temporally associated with preoperative GnRH agonist therapy. The subsequent clinical course showed marked regression and calcification of a small residual intramural component, with complete clearance of the endometrial cavity confirmed by hysteroscopy. Postpartum uterine involution, altered vascular dynamics, fibroid infarction, and myometrial contractility were likely important contributing factors, while leuprorelin acetate may have facilitated degeneration and expulsion. As causality cannot be established from a single case, the findings should be interpreted cautiously. Careful clinical, imaging, hysteroscopic, and histological follow-up is essential to confirm endometrial cavity clearance, exclude residual intracavitary or atypical pathology, and identify potential complications.

## References

[1] Baird DD, Dunson DB, Hill MC, Cousins D, Schectman JM (2003). High cumulative incidence of uterine leiomyoma in black and white women: ultrasound evidence. Am J Obstet Gynecol.

[2] Schwartz SM, Marshall LM, Baird DD (2000). Epidemiologic contributions to understanding the etiology of uterine leiomyomata. Environ Health Perspect.

[3] Sinai Talaulikar V (2018). Medical therapy for fibroids: an overview. Best Pract Res Clin Obstet Gynaecol.

[4] Kashani BN, Centini G, Morelli SS, Weiss G, Petraglia F (2016). Role of medical management for uterine leiomyomas. Best Pract Res Clin Obstet Gynaecol.

[5] Vanderhoff AC, Silberman J, Gargiulo AR (2022). Current trends in the evaluation and management of uterine fibroids. Curr Obstet Gynecol Rep.

[6] Kessel B, Liu JH, Mortola JF, Berga SL, Yen SS (1988). Treatment of uterine fibroids with agonist analogs of gonadotropin-releasing hormone. Fertil Steril.

[7] Friedman AJ, Lobel SM, Rein MS, Barbieri RL (1990). Efficacy and safety considerations in women with uterine leiomyomas treated with gonadotropin-releasing hormone agonists: the estrogen threshold hypothesis. Am J Obstet Gynecol.

[8] Adamson GD (1992). Treatment of uterine fibroids: current findings with gonadotropin-releasing hormone agonists. Am J Obstet Gynecol.

[9] Bifulco G, Miele C, Pellicano M (2004). Molecular mechanisms involved in GnRH analogue-related apoptosis for uterine leiomyomas. Mol Hum Reprod.

[10] Maruo T, Ohara N, Wang J, Matsuo H (2004). Sex steroidal regulation of uterine leiomyoma growth and apoptosis. Hum Reprod Update.

[11] De Cure N, Sullivan T, Robertson M, Hallam L, Whale K (2013). Spontaneous expulsion of large submucosal uterine fibroid without embolisation - a case study. Australas J Ultrasound Med.

[12] Honoré LH, Reid DW (1985). Uncomplicated, spontaneous expulsion of a uterine leiomyoma postpartum. A case report. J Reprod Med.

[13] Haskins RD Jr, Haskins CJ, Gilmore R, Borel MA, Mancuso P (2001). Intramural leiomyoma during pregnancy becoming pedunculated postpartally. A case report. J Reprod Med.

[14] Kim M (2018). Spontaneous complete regression of large uterine fibroid after the second vaginal delivery: case report. Medicine (Baltimore).

[15] Zhang J, Zou B, Wang K (2018). Spontaneous expulsion of a huge cervical leiomyoma from the vagina after cesarean: a case report with literature review. Medicine (Baltimore).

[16] Yu KJ, Lai CR, Sheu MH (2001). Spontaneous expulsion of a uterine submucosal leiomyoma after administration of a gonadotropin-releasing hormone agonist. Eur J Obstet Gynecol Reprod Biol.

[17] Wen L, Tseng JY, Wang PH (2006). Vaginal expulsion of a submucosal myoma during treatment with long-acting gonadotropin-releasing hormone agonist. Taiwan J Obstet Gynecol.

[18] Atilgan R, Simsek M, Ozkan ZS (2013). Spontaneous myomectomy after GnRH agonist treatment: case report. Turkiye Klinikleri J Gynecol Obst.

[19] Ishizawa C, Hirota Y, Urata Y, Morishima K, Fujii T, Osuga Y (2020). Prolapse of a pedunculated uterine leiomyoma through the cervix during GnRH antagonist treatment: case report and literature review. J Obstet Gynaecol Res.

